# Should Preachers Pay?

**Published:** 1887-06

**Authors:** Ross P. Cox

**Affiliations:** Rome, Ga.


### SHOULD PREACHERS PAY?

*Editors Atlanta Medical and Surgical Journal:*

The question that introduces this article has received in recent numbers of THE JOURNAL a spirited agitation at the hands of West and Kirwan.

Although Dr. West, who represents the affirmative, perhaps advanced some views that are illy considered and untenable, few, doubtless, would deny that Kirwan's assertion that West's presentations are " alleged facts that are as unreal as the mockery of a vision, or the vague fancies of a sick man's dream," is both inconsequential and incorrect. Speaking in the light of what has been learned from physicians, clergymen and disinterested persons, the statement might safely be ventured that the discerning, unprejudiced mind must subscribe to the editorial utterance of the *Medical Record*, that " the arguments put forward (by West) are in the main sensible and sound."

"Kirwan" seems to be especially astounded and grieved that any one should have the impious boldness to attack " time-honored and well-nigh universal customs." He seems deeply imbued with the philosophy of "it is, and therefore ought to be." One cannot but think how noble a champion of " passive obedience " and " divine rights of kings " he would have been had he flourished during the seventeenth century.

He should know that a new thing may now and then be a good one. Though now, doubtless, one of the "precious factors in our Christian civilization," it was once a "time-honored and well-nigh universal custom" to rate such men very differently. May he not, even though in this minor matter, have fallen into an error which differs only in degree from that which, ever since the birth of time, has made good the statement that "every pillar in the great temple of Truth rests on the grave of a martyr?"

Yet, no one can deny that antiquity and universality of customs in vogue afford *prima facie* evidence of their desirability and utility. Nevertheless, although it probably would not be suicidal to grant my facetious opponent the full benefit of the argument that was used against Galileo, Luther, Harvey and Jenner, yet there is no necessity for so doing.

It is a matter of fact that some physicians do exact fees for services from such of the clergy as are able to pay them. I challenge your correspondent of doubtful identity to produce, either from the American Code of Medical Ethics or that of Great Britain, statements that authorize, or even permit, such a system of clerical deadheadism as he advocates. The American Code says: "Poverty, professional brotherhood and certain of the public duties referred to in the first section of this article should always be recognized as presenting valid claims for gratuitous services, but neither institutions endowed by the public, or by rich individuals, societies for mutual benefit, for the insurance of lives or for analogous purposes, *nor any profession* or occupation can be admitted to possess such privileges."

The following query and reply, taken from a recent number of the *British Medical Journal*, speaks for itself: "Sir—I have just received the subjoined, 'Dear Dr. ——, I have been so frequent a patient of yours lately that I am obliged to ask a question that has occurred to me before: Is it not the custom in the profession to attend the local clergy either gratuitously or at reduced rates? Yours, very truly, etc.' Will you please state in your next issue your opinion on the point raised?" "I may say that my own rule, for the last fifteen years, has been to attend gratuitously all underpaid incumbents and all curates (unless they were men of private means), but to charge ordinary incumbents as ordinary patients." "The writer of the above is an unmarried vicar, whose income, according to the clergy list, is £500 a year. I am yours truly, X. Y. Z."

The editor replies: "'X. Y. Z.' has, we opine, been somewhat of an unobservant, or, mayhap, a non-reader of our answers to correspondents, or he could scarcely have failed to observe that the point raised in his note has been answered in our columns again and again. Nevertheless, we willingly accede to his request and quote for his guidance the following parenthetical extract from the Code of Medical Ethics, second edition, chap. II, sec. 7, page 79: In respect to charges for professional attendance on the clergy, beneficed or unbeneficed, and their families, there is no special general rule other than the simple 'unwritten' one (a time-honored and 'true Samaritan' principle alike applicable to those classes) by which the faculty have long been self-guided, namely, although fully and justly entitled to a commensurate remuneration for professional services accordant to the patient's position in life, nevertheless to make a greater or less reduction, according to the circumstances of the individual case to such as may fairly be classed among the 'poor clergy' (beneficed or unbeneficed), specially so-called in contradistinction to the well-endowed and independent clergy, which latter should be charged as ordinary and not exceptional patients."

The editor adds: "That an unmarried beneficed clergyman, with an income of £500 a year, should seek eleemosynary (for it is nothing less) medical aid from a practicing member of a hard-worked and under-paid profession is, to our mind, 'passing strange.'"

These facts appear to shift the burden of proof somewhat.

"Kirwan," speaking of West's demands, says: "In its last analysis it is the 'pound of flesh' which Shylock demanded, because it was so nominated in the bond. It is not strange that any one who thus metaphorizes the moderate fee which the physician asks for his services should take refuge behind a pseudonym."

That a bond of sympathy and good will should exist between all men who are striving to ameliorate the human lot and elevate the lives and aspirations of men is a fact not likely to escape the notice of any one revolving the subject. Journalists, authors, educators and philanthropists everywhere, as well as ministers and physicians, to a considerable extent at least, are exemplifying the principles of an universal brotherhood and do a vast deal of unrequited work for the benefit of their fellows.

But, while sentiments of high esteem and exalted friendship should, and often do, prevail among such men, is it just or reasonable to require that the simple and necessary business principles that guide civilized men everywhere should be suspended in their dealings one with the other? A *reductio ad absurdum* would be as easy along here as Kirwan supposes it is against the view he combats.

With regard to physicians charging each other, it is sufficient to say it is customary for the members of every guild to show favors to each other not extended to others, and when the physician makes no charges there is generally the idea of a return of the favor, which reciprocity cannot be expected from those outside his profession.

I cannot think so meanly of the Christian ministry as to think many of its members propose to pay their medical bills by helping their physician into practice among the members of their several flocks. To do so would be little else than trafficing in their influence and selling it for a price.

Perhaps the reasons why so many well-to-do, influential ministers have been willing to accept unpaid medical aid are that they believe the custom "time-honored" and general. They found it convenient and inexpensive, and it accorded with a greater or less streak of the Peter Mullens in them. Perhaps the reasons why physicians have without recompense rendered services to ministers of means and influence are motives of policy and senseless continuation and extension of a custom which probably was at first based in reason and justice.

*Rome, Ga.* ROSS P. COX.

